# Validated liquid chromatography tandem mass spectrometry for simultaneous quantification of foretinib and lapatinib, and application to metabolic stability investigation

**DOI:** 10.1039/c9ra03251g

**Published:** 2019-06-20

**Authors:** Mohammed M. Alanazi, Hamad M. Alkahtani, Abdulrahman A. Almehizia, Mohamed W. Attwa, Ahmed H. Bakheit, Hany W. Darwish

**Affiliations:** Department of Pharmaceutical Chemistry, College of Pharmacy, King Saud University P.O. Box 2457 Riyadh 11451 Saudi Arabia mehizia@ksu.edu.sa mzeidan@ksu.edu.sa +966 1146 76 220 +966 1146 70237; Students' University Hospital, Mansoura University Mansoura 35516 Egypt; Department of Chemistry, Faculty of Science and Technology, Al-Neelain University Khartoum Sudan; Analytical Chemistry Department, Faculty of Pharmacy, Cairo University Kasr El-Aini St. Cairo 11562 Egypt

## Abstract

Foretinib (GSK1363089, FTB) is a multikinase inhibitor that inhibits multiple receptor tyrosine kinases, including vascular endothelial growth factor receptor-2 and mesenchymal–epithelial transition factor, with the potential for solid tumor treatment. Lapatinib (LPB) is a significant promising drug molecule that was approved by the USFDA and was utilized to develop a nontoxic and very efficient targeted therapy against breast cancer. There is an ongoing clinical trial for using of FTB and LPB combination for HER-2 positive metastatic breast cancer treatment. In the current study, liquid chromatography tandem mass spectrometry methodology was validated for simultaneous estimation of FTB and LPB with application to drug metabolic stability investigation. Chromatographic separation of FTB, LPB and masitinib (internal standard) was attained using an isocratic mobile phase running on a reversed-phase C_18_ column. The linear dynamic range was 5–500 ng mL^−1^ with *r*^2^ ≥ 0.9999 in the rat liver microsomes (RLMs) matrix. The FTB and LPB metabolic stabilities in the RLMs matrix were estimated by computing two parameters, intrinsic clearance (CL_int_: 6.33 and 5.63 mL min^−1^ kg^−1^) and a low *in vitro* half-life (*t*_1/2_: 23.9 and 26.9 min), which revealed the FTB and LPB high clearance by the liver from the blood. This probably revealed the low *in vivo* bioavailability that verified the low oral bioavailability previously reported and also indicated that FTB and LPB will not bioaccumulate after multiple doses. FTB metabolic rate is slightly decreased in combination with LPB, while LPB metabolic rate is greatly increased in combination with FTB. So dose recalculation must be evaluated when FTB and LPB are used in combination.

## Abstract 2

Foretinib metabolic rate is decreased in combination with lapatinib. Lapatinib metabolic rate is greatly increased in combination with foretinib. Dose recalculation should be considered when foretinib and lapatinib are used in combination.

## Graphical abstract

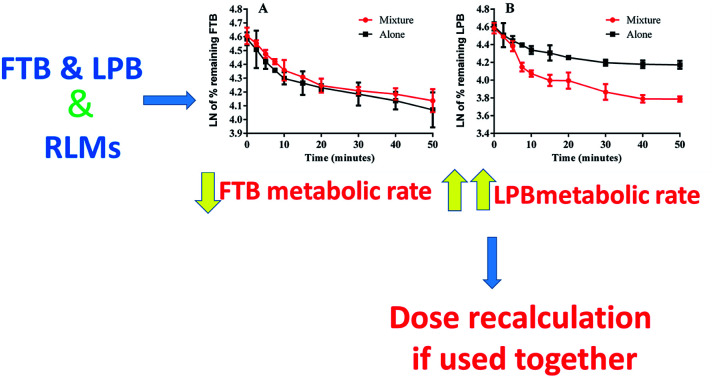

## 1. Introduction

Tyrosine kinase inhibitors (TKIs) are a class of drugs that inhibit the intracellular signals that drive proliferation in numerous malignant cells by unambiguously inhibiting the kinase activities of distinct intracellular pathways involved in receptor mediated growth signaling.^1,2^ Tyrosine kinases are enzymes which control γ phosphate group of ATP transfer to the tyrosine hydroxyl groups on marked proteins. They play as “on” or “off” switch in various cellular functions.^3^ Firm management of the tyrosine kinase activity in the cell controls important processes such as proliferation, cell cycle and cell death. In several cases, the cancer abnormal proliferation characteristics are generated by growth factor receptor-mediated signaling. In tumor cells, the failure of the control mechanism may initiate excessive phosphorylation, and pathways retained in an activated state.^4,5^ Foretinib (FTB) and lapatinib (LPB) are TKIs.

**Fig. 1:**
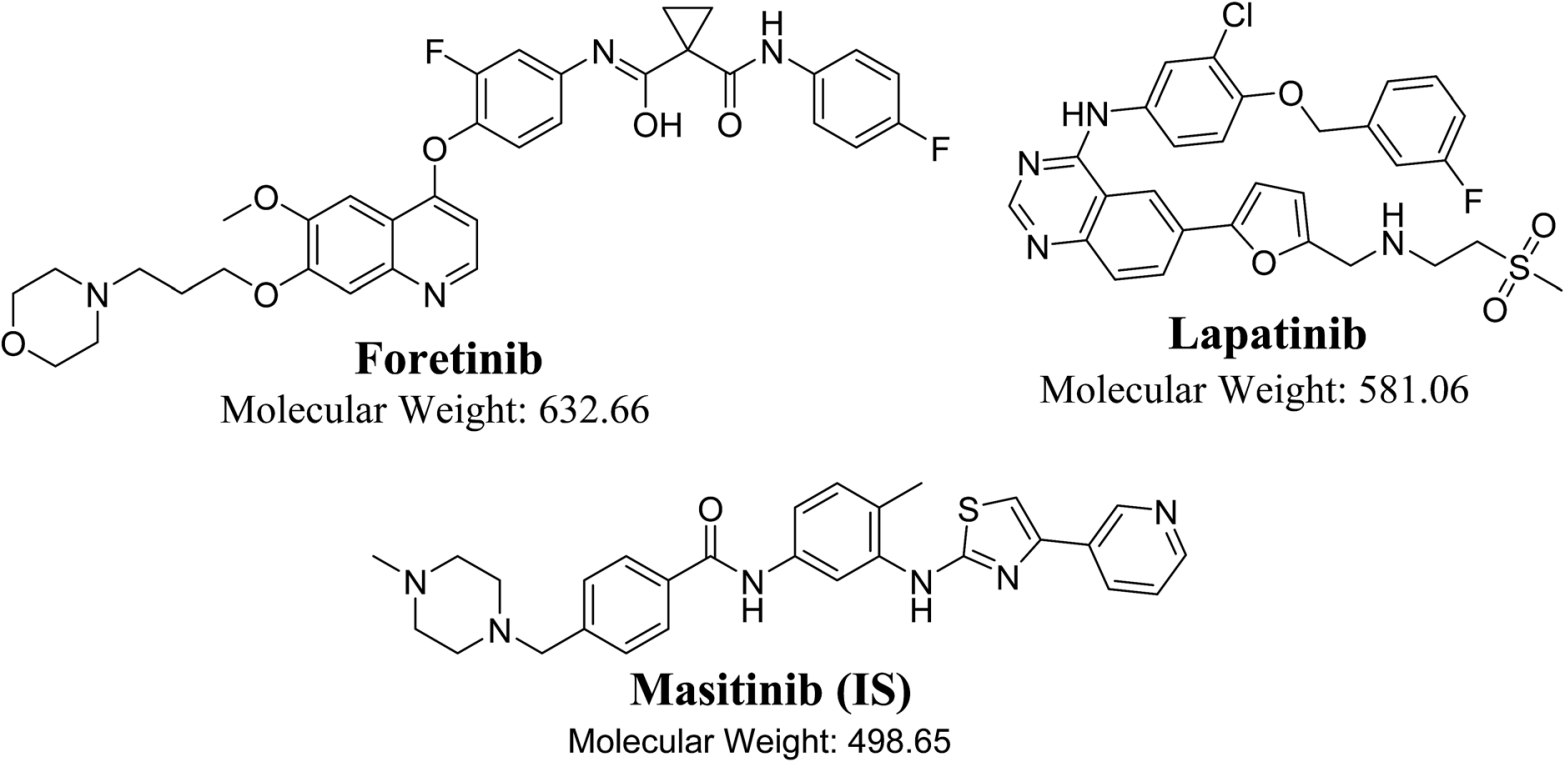
Chemical structures of foretinib, lapatinib and masitinib (IS).

FTB (GSK1363089, Fig. 1) is a multikinase inhibitor that inhibits multiple receptor tyrosine kinases, including vascular endothelial growth factor receptor-2 (VEGFR) and mesenchymal–epithelial transition factor (MET), with the capability for solid tumors treatment.^6^ GlaxoSmithKline launched many clinical trials for FTB in many types of cancers but in 2015, it suddenly discontinued their clinical studies for FTB. LPB (Fig. 1) is a significant promising drug molecules, approved by FDA, USA is being utilizing for the development of a nontoxic and effective targeted therapy against breast cancer.^7^ The LPB clinical effectiveness in combination with capecitabine has exhibited efficacy contrary to HER2-positive breast cancer.^8^ Besides, LPB-loaded human serum albumin nano particles have been proposed as a nontoxic therapy against HER2-positive cells.^9^ LPB belongs to a class of tyrosine kinase inhibitor, is hydrophobic in nature. LPB suppresses the abnormal activity of HER2 and EGFR by inhibiting their phosphorylation.^10^ Even though, LPB has exhibited 99% bound to α-1 acid glycoprotein and human serum albumin in the blood stream.^11^

There is an undergoing clinical trial for the use of FTB and LPB combination for the treatment of HER-2 positive metastatic breast cancer.^12^ To the best of our knowledge, there has been no published analytical method for simultaneous estimation of FTB and LPB. Therefore, the purpose of the present study was to establish a validated LC-MS/MS method to quantify FTB and LPB with application to metabolic stability estimation by computing CL_int_*in vitro* half-life (*t*_1/2_). These parameters could then be used for bioavailability calculations, hepatic clearance and *in vivo t*_1/2_. Bioavailability is crucial as it provides an idea for tested drug metabolism; if the drug is rapidly metabolized, it will showed low bioavailability *in vivo*.^13^ Also studying the combined form metabolic stability may gave an idea about the drug–drug interaction between FTB and LPB.

## 2. Experimental

### 2.1. Chemicals and reagents

All reagents and solvents were of analytical grade. Lapatinib was purchased from Med Chem Express (USA). Foretinib (FTB) and masitinib (MST) were purchased from LC Laboratories (USA). Acetonitrile (ACN), Rat liver microsomes (RLMs), and formic acid (HCOOH) were purchased from Sigma-Aldrich (USA). HPLC-grade water was obtained from the Milli-Q plus purification system (Millipore, USA).

### 2.2. Liquid chromatography tandem mass spectrometry methodology

**Scheme 1:**
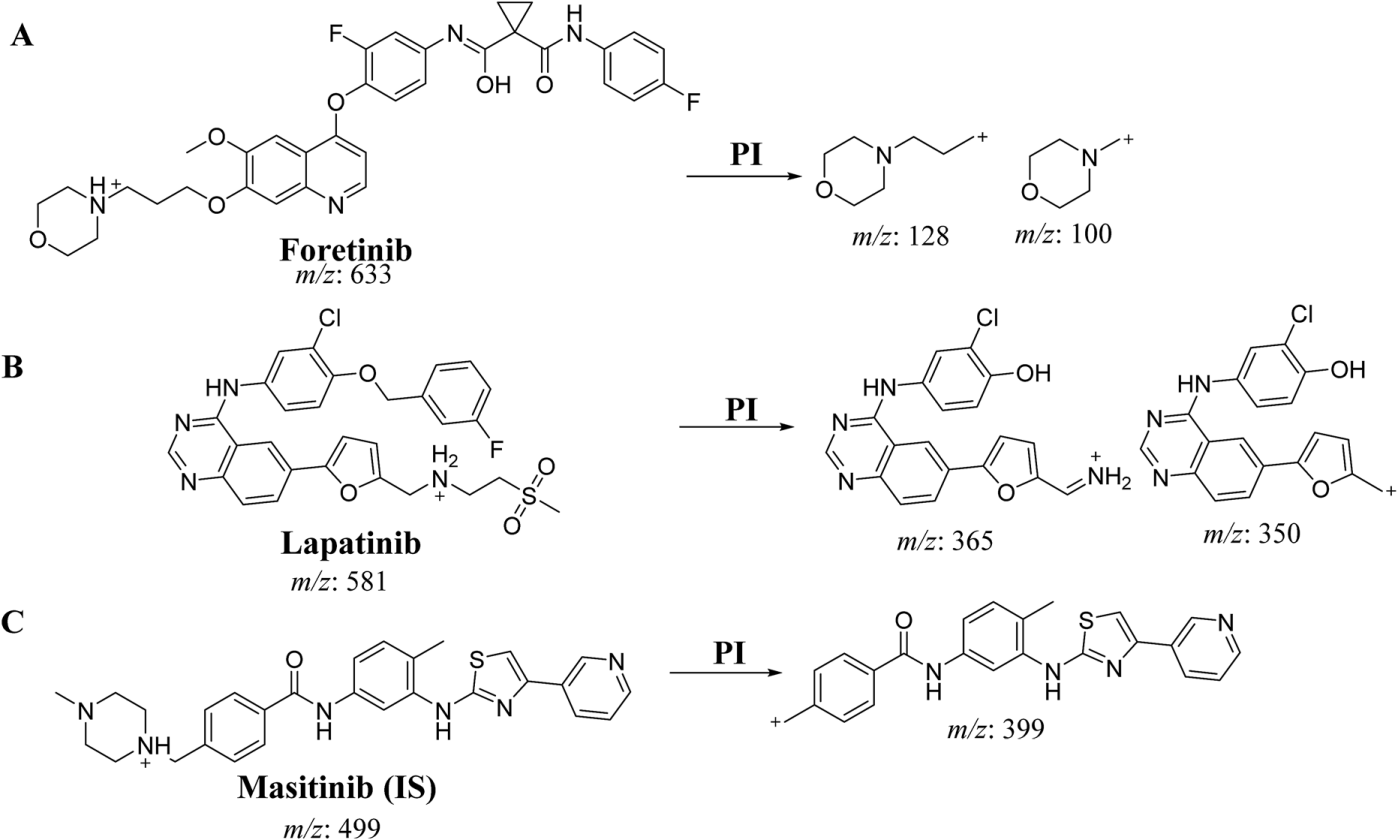
Product ion of FTB (A), LPB (B) and MST (C).

An Acquity UPLC [model code (UPH) and serial number (H10UPH)] was used chromatographic separation of RLMs incubates while Acquity TQD MS [model code (TQD) and serial number (QBB1203)] was used for mass analysis of eluted analytes peaks. MST was selected as the IS in the FTB and LPB analysis as the same method of extraction could be applied for both MST, FTB and LPB. MST is considered a TKIs and are not reported to be administered together with either FTB or LPB to the same patient at the same time. Fragmentation pattern for the analytes were explained in Scheme 1. All LC-MS/MS parameters were adjusted for efficient and fast separation for tested analytes (Table 1). Multiple reaction monitoring (MRM) detection mode was used in mass analysis of LPB, FTB and IS (Scheme 1).

**Table 1:** Optimized parameters of LC-MS/MS method

Parameters of Acquity UPLC		Parameters of Acquity TQD MS	
Isocratic mobile phase	45% ACN	Ionization source	Positive ESI
	55% aqueous (10 mM ammonium formate in H_2_O (pH: 4.2 adjusted by adding few drops of formic acid))		Drying gas: N_2_ gas
	Flow rate: 0.25 mL min^−1^		Flow rate (12 L min^−1^)
	Injection volume: 5 μL		Pressure (60 psi)
ACQUITY UPLC BEH C18 column at *T*: 22 ± 1 °C	50 mm in length		Source temperature: 350 °C
	130 Å pore size		Capillary voltage: 4000 V
	2.1 mm in internal diameter	Collision cell gas	Nitrogen with high purity
	1.7 μm particle size	Mode	Multiple reaction monitoring (MRM)
Analyte	Lapatinib (LPB)	LPB MRM transitions	*m*/*z* 581 → *m*/*z* 350, CO^a^: 62 V, CE^b^: of 38 eV
			*m*/*z* 581 → *m*/*z* 365, CO^a^: 62 V, CE: of 38 eV
	Foretinib (FTB)	FTB MRM transitions	*m*/*z* 633 → *m*/*z* 100, CO^a^: 46 V, CE^b^: of 34 eV
			*m*/*z* 633 → *m*/*z* 128, CO^a^: 46 V, CE: of 32 eV
IS	Masitinib (MST)	MST MRM Transitions	*m*/*z* 499 → *m*/*z* 399.06 CO^a^: 42 V, CE: 24 eV

a Cone voltage.

b Collision energy.

### 2.3. Standard solutions of FTB and LPB

FTB, LPB and MST are dimethyl sulfoxide soluble. Stock of FTB and LPB were prepared (2 mg mL^−1^) followed by ten times dilution with mobile phase to prepare FTB S1 (200 μg mL^−1^) and LPB S1 (200 μg mL^−1^), which was then further diluted ten times with mobile phase to prepare FTB S2 (20 μg mL^−1^) and LPB S2 (20 μg mL^−1^). Stock of masitinib solution (100 μg mL^−1^) was prepared in dimethyl sulfoxide and then further diluted fifty times with the mobile phase to prepare working MST S3 (2 μg mL^−1^).

### 2.4. Preparation of calibration standards

LPB S2 (20 μg mL^−1^) and FTB S2 (20 μg mL^−1^) were diluted with specific RLMs matrix (40 μL in 1 mL of phosphate buffer) to generate twelve calibration standards: 5, 10, 15, 30, 50, 75, 100, 150, 200, 300, 400, and 500 ng mL^−1^. Three standards (15, 150, and 400 ng mL^−1^) were selected as quality controls, namely the low quality control (LQC), medium quality control (MQC), and high quality control (HQC), respectively. Fifty microliters of MST S3 was added to 1 mL of each calibration standard.

### 2.5. FTB and LPB extraction from RLMs matrix

Analytes extractions were done using ACN protein precipitation, a standard technique for metabolic stability experiments.^14–17^ ACN was used for precipitation followed by centrifugation (14 000 rpm, 12 min at 4 °C) then supernatants were filtered using syringe filters (0.22 μm pore size). Filtered samples were transferred to 1.5 mL vials. Five μL of each sample was then injected into the LC-MS/MS for analysis. Similarly, blank samples were prepared by using the stated phosphate buffer without RLMs matrix to confirm that RLMs components did not interfere with the elution time of analytes. Two calibration curve were established by plotting the peak area ratio of LPB to MST (*y* axis) against the nominal values (*x* axis) and the peak area ratio of FTB to MST (*y* axis) against the nominal values (*x* axis). A linear regression equation was used to validate the linearity of the established method. Slope, intercept, and coefficient of determination (*r*^2^) values were computed.

### 2.6. Method validation

Validation parameters of the established LC-MS/MS method were described in more detail previously^15,18,19^ that included assay recovery, sensitivity, specificity, linearity, reproducibility, limit of detection (LOD) and limit of quantification (LOQ). The least squares statistical method was used to calculate the calibration curve equations (*y* = *ax* + *b*). The linear fit was verified using the *R*^2^ value, which was linear in the range from 2–500 ng mL^−1^.

### 2.7. Metabolic stability of LPB and FTB

The metabolic stability study for LPB and FTB were done by evaluating the decrease in LPB and FTB concentrations after incubation with RLMs. One μM FTB and 1 μM LPB were incubated with RLMs (1 mg protein) in triplicate. The metabolic reaction medium was phosphate buffer (pH 7.4) that contains 3.3 mM MgCl_2_. The metabolic mixture was pre-incubated at 37 °C water bath for temperature conditioning for 10 min. NADPH (1 mM) was used to initiate the metabolic reaction while 2 mL ACN was used to terminate it at specific time intervals (0, 2.5, 5, 7.5, 10, 15, 20, 40, and 50 min). The metabolic stability curve for FTB and LPB were then created. The same metabolic reaction was repeated for each analyte (LPB and FTB) in a separate experiment to assess the drug metabolic stability alone or in a mixture of both.

## 3. Results and discussion

### 3.1. HPLC–MS/MS methodology

**Fig. 2:**
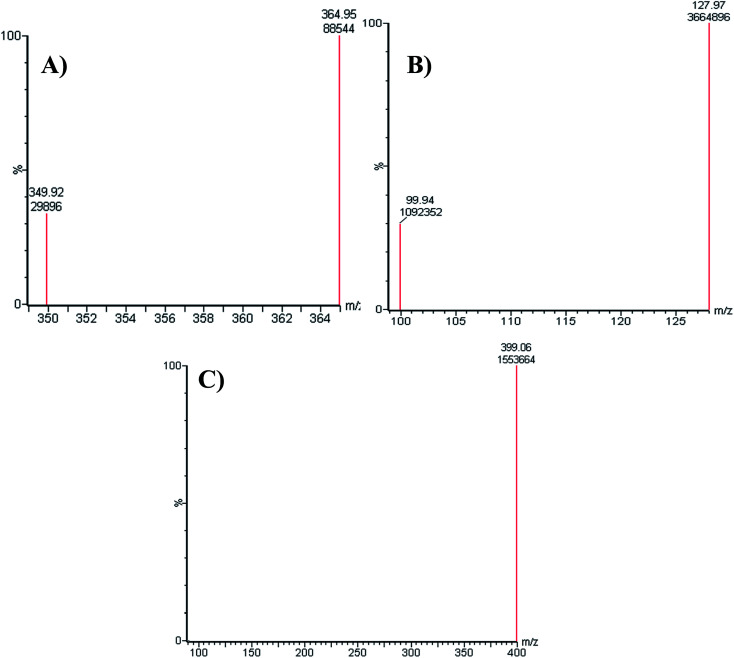
MRM mass spectrum transitions of LPB (A), FTB (B) and MST (C).

All chromatographic parameters including the mobile phase pH, mobile phase constituents, and C_18_ column were adjusted. The aqueous mobile phase part pH (10 mM NH_4_COOH) was adjusted to 4.2 with formic acid as above this value caused unnecessary increase in elution time and peak tailing. The aqueous to organic (ACN) ratio parts of the mobile phase was optimized at 45% : 55% as ACN increase generated overlapped chromatographic peaks with bad resolution and ACN decrease resulted in higher elution times. Various types of stationary phases (*e.g.*, HILIC columns) were checked, but LPB, FTB and MST were not separated. The perfect results were attained using C_18_ columns. The MRM mode of the mass analyzer was utilized for LPB and FTB ions estimation to avoid potential interference from the RLM matrix constituents and increase the sensitivity of the established LC-MS/MS method (Fig. 2).

**Fig. 3:**
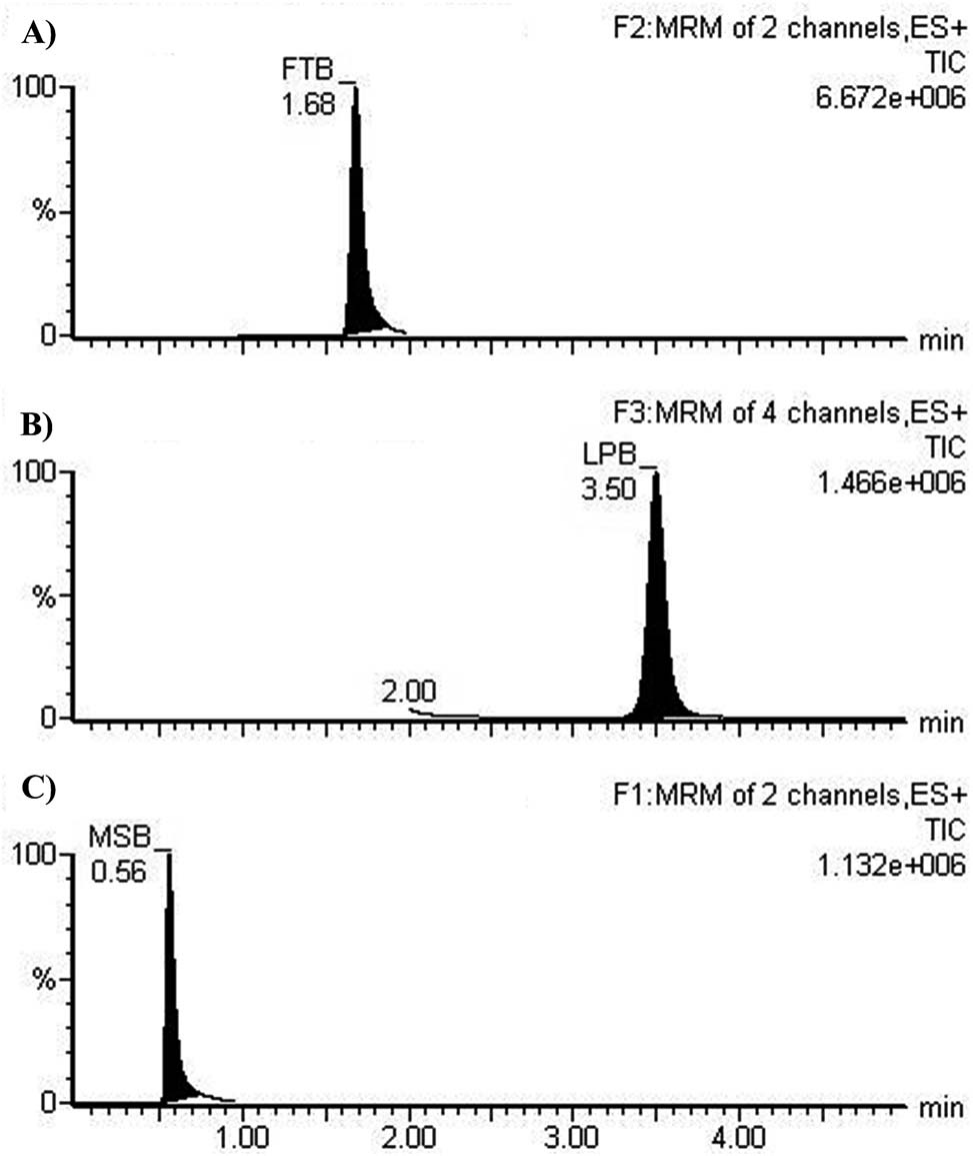
MRM chromatograms of (A) FTB at 1.68 min, (B) LPB at 3.5 min. and (C) MST at 0.56 min.

The chromatographic resolution of LPB, FTB and MST were attained in 5 min with good resolution (Fig. 3).

### 3.2. Method validation of the developed LC-MS/MS method

#### 3.2.1. Specificity

MRM chromatograms reveal good resolution of the LPB, FTB and IS peaks and the absence of peaks with the blank RLM matrix at their elution times, which approves the specificity of the established methodology (Fig. 3).

#### 3.2.2. Sensitivity and linearity

The regression line linear range and correlation coefficient (*r*^2^) for the developed chromatographic method were 5–500 ng mL^−1^ and ≥ 0.9997 in the RLM matrix, respectively. The FTB calibration curve regression equation was *y* = 0.01925*x* + 0.2236. LOD and LOQ for FTB calibration curve were equal to 1.2 and 3.64 ng mL^−1^, respectively. The LPB calibration curve regression equation was *y* = 0.004721*x* + 0.03558. LOD and LOQ for LPB calibration curve were equal to 2.66 and 8.051 ng mL^−1^, respectively. The RSD values of six replicates for each standard level in the calibration curve were less than 4.93% and 3.96% (FTB and LPB) in the RLM matrix (Table 2). Back calculations of the fifteen standards of LPB in the RLM matrix (calibration and QC standards) approved the performance of the developed methodology.

**Table 2:** Data of LPB and FTB back-calculated concentration of the calibration levels from RLMs matrix

Nominal concentrations in ng mL^−1^	LPB				FTB			
	Mean^a^	SD	RSD%	Accuracy%	Mean^a^	SD	RSD%	Accuracy%
5	4.81	0.16	3.39	96.19	4.96	0.22	4.48	99.30
10	9.51	0.29	3.06	95.12	10.33	0.51	4.93	103.26
15	15.01	0.59	3.96	100.04	15.6	0.26	1.68	104.03
30	30.39	1.04	3.41	101.29	29.7	0.83	2.79	99.00
50	49.58	0.39	0.78	99.16	49.33	1.96	3.97	98.65
75	74.83	0.55	0.74	99.78	73.51	2.31	3.14	98.01
100	100.57	1.16	1.15	100.57	97.69	3.05	3.12	97.69
150	147.41	5.73	3.88	98.27	147.77	3.51	2.38	98.51
200	203.89	2.95	1.44	101.95	196.66	6.48	3.29	98.33
300	301.54	1.75	0.58	100.51	300.88	8.68	2.89	100.29
400	396.32	1.42	0.36	99.08	402.22	3.83	0.95	100.56
500	501.25	2.39	0.48	100.25	508.61	17.13	3.37	101.72
% recovery				99.35				99.95
SD				2				2.08

a Average of six replicates.

#### 3.2.3. Precision and accuracy

The intra- and inter-day accuracy and precision values are acceptable according to International Council for Harmonisation (ICH) guidelines (Table 3)^20,21^ as they are ranged from 0.36 to 3.96% and 95.12 to 101.95% for LPB, respectively, and ranged from 0.95 to 4.93% and 97.69 to 104.03% for FTB, respectively (Table 3).

**Table 3:** Precision and accuracy (intra-day and inter-day) of the developed methods

RLMs matrix			Mean	SD	Precision (% RSD)	% accuracy
FTB	LQC (15.00 ng mL^−1^)	Intra-day assay^a^	16.09	0.44	2.71	107.27
		Inter-day assay^b^	16.02	0.28	1.77	106.81
	MQC (150.00 ng mL^−1^)	Intra-day assay^a^	147.14	3.71	2.52	98.09
		Inter-day assay^b^	148.52	6.98	4.70	99.01
	HQC (400.00 ng mL^−1^)	Intra-day assay^a^	400.00	7.17	1.79	100.00
		Inter-day assay^b^	400.95	7.30	1.82	100.24
LPB	LQC (15.00 ng mL^−1^)	Intra-day assay^a^	15.19	1.05	6.90	101.27
		Inter-day assay^b^	15.58	0.50	3.19	103.88
	MQC (150.00 ng mL^−1^)	Intra-day assay^a^	141.77	5.17	3.64	94.51
		Inter-day assay^b^	144.75	7.10	4.91	96.50
	HQC (400.00 ng mL^−1^)	Intra-day assay^a^	395.91	3.28	0.83	98.98
		Inter-day assay^b^	400.26	6.20	1.55	100.07

a Average of twelve replicates of day 1.

b Average of six replicates in three consecutive days.

#### 3.2.4. Matrix effects and extraction recovery

Table 4 shows the recovery percentages of the quality controls when computing FTB and LPB concentration with the RLMs matrix. The FTB and LPB recoveries were 101.7 ± 4.8%, 98.6 ± 2.3%, respectively in the RLMs matrix. The RLMs matrix effect absence was verified by analyzing six various batches of RLMs matrixes form six different rats. These batches (named set 1) were extracted then spiked with FTB and LPB LQCs (15 ng mL^−1^) and MST (IS). Set 2 batches were prepared in the same way utilizing the mobile phase instead of the RLMs matrix. Thus, the matrix effect was computed using the next equation:
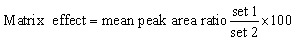


**Table 4:** Recovery of QC samples in RLMs matrix

	RLMs matrix					
	FTB			LPB		
Nominal concentration (ng mL^−1^)	15 ng mL^−1^	150 ng mL^−1^	400 ng mL^−1^	15 ng mL^−1^	150 ng mL^−1^	400 ng mL^−1^
Mean^a^	16.08	147.22	399.41	14.4	148.87	401.92
Recovery (%)	107.23	98.15	99.85	95.97	99.25	100.48
SD	0.68	5.78	11.19	0.67	1.84	2.47
Precision (RSD%)	4.23	3.93	2.8	4.66	1.24	0.62

a Average of six replicates.

The tested RLM matrix that contained FTB and LPB had a matrix effect of 99.7 ± 4.8% and 97.9 ± 3.6%, respectively. Accordingly, these outcomes approved that there is very low influence of the RLM matrix on FTB, LPB and MST (IS) ionization.

#### 3.2.5. Stability

FTB and LPB stabilities of in RLMs matrix were tested under all laboratory conditions that may have been subjected before drug analysis. FTB and LPB exhibited good stability in RLMs matrix following storage at −20 °C for 30 days. Measured values were ranged from 95.45 to 107.6% for FTB and from 85.6 to 105.9% for LPB. FTB and LPB stability data is mentioned in Table 5. There was no noticeable degradation of analytes under the examined conditions.

**Table 5:** Stability data of FTB and LPB in RLMs matrix under different conditions

	Nominal concentration (ng mL^−1^)	Mean (ng mL^−1^)	Recovery%	Precision (RSD%)
FTB	Room Temp. for 8 h			
	15	15.33 ± 0.75	102.2	4.92
	150	148.78 ± 7.77	99.19	5.22
	400	401.25 ± 8.12	100.31	2.02
	Three freeze–thaw cycles			
	15	14.56 ± 0.42	97.09	3.64
	150	150.96 ± 5.00	100.64	2.38
	400	399.48 ± 6.25	99.87	1.01
	Stored at 4 °C for 24 h			
	15	14.32 ± 0.60	95.45	4.2
	150	148.85 ± 3.67	99.24	2.46
	400	399.11 ± 3.20	99.78	0.80
	Stored at −20 °C for 30 days			
	15	16.13 ± 0.18	107.55	1.11
	150	146.50 ± 6.77	97.67	4.62
	400	397.94 ± 3.84	99.49	0.97
LPB	Room Temp. for 8 h			
	15	15.8 ± 0.28	105.34	1.77
	150	145.16 ± 1.81	96.77	1.24
	400	401.69 ± 2.02	100.42	0.5
	Three freeze–thaw cycles			
	15	15.43 ± 0.95	102.86	6.15
	150	145.75 ± 2.29	97.17	1.57
	400	401.5 ± 3.13	100.37	0.78
	Stored at 4 °C for 24 h			
	15	15.89 ± 0.31	105.92	1.93
	150	137.57 ± 2.39	91.72	1.73
	400	396.36 ± 6.46	99.09	1.63
	Stored at −20 °C for 30 days			
	15	14.13 ± 0.44	94.23	3.13
	150	128.43 ± 1.60	85.62	1.25
	400	355.92 ± 1.79	88.98	0.5

### 3.3. Metabolic stability

**Fig. 4:**
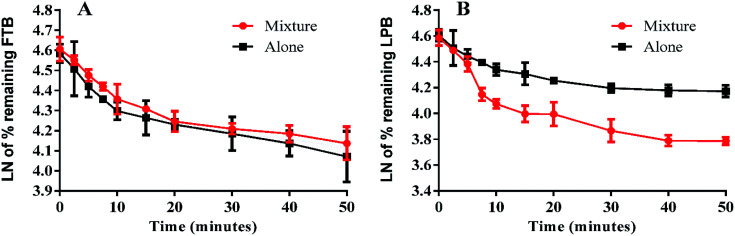
Metabolic stability curve of FTB alone or in a mixture with LPB (A). Metabolic stability curve of LPB alone or in a mixture with FTB (B).

The LPB and FTB concentrations in the RLMs matrix were computed by substitution of the peak area ratios in the linear calibration curve regression equation. Metabolic stability curves were drawn for FTB and LPB alone or in a mixture (Fig. 4). The linear portion of the established curve was used to compute *in vitro t*_1/2_.^22^ The regression equations for this linear region were for FTB (alone: *y* = −0.029*x* + 4.5784 with *R*^2^ = 0.994, mix: *y* = −0.0251*x* + 4.6073 with *R*^2^ = 0.998) and for LPB (alone: *y* = −0.0258*x* + 4.5867 with *R*^2^ = 0.979, mix: *y* = −0.0546*x* + 4.6088 with *R*^2^ = 0.969) (Table 6).

**Table 6:** Metabolic stability parameters for FTB and LPB incubations with RLMs

	FTB metabolic stability parameters		LPB metabolic stability parameters	
	Alone	Mixture	Alone	Mixture
Regression equation^a^	*y* = −0.029*x* + 4.5784	*y* = −0.0251*x* + 4.6073	*y* = −0.0258*x* + 4.5867	*y* = −0.0546*x* + 4.6088
*R* ^2^ b	^b^0.9935	0.9977	0.979	0.9695
Slope	0.029	0.0251	0.0258	0.0546
*t*_1/2_ c	23.9 min	27.6 min	26.9 min	12.7 min
CL_int_^d^	6.33 mL min^−1^ kg^−1^	5.48 mL min^−1^ kg^−1^	5.63 mL min^−1^ kg^−1^	11.91 mL min^−1^ kg^−1^

a Regression equation of linear portion of curve.

b Correlation coefficient.

c Half-life.

d Intrinsic clearance.

The slope for each regression equation was substituted in the next equations:*In vitro t*_1/2_ = ln2/slope*In vitro t*_1/2_ = 0.693/slope

The intrinsic clearance (CL_int_) of LPB and FTB were computed using the *in vitro t*_1/2_ method^13^ as in the following equation:
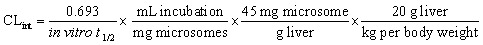

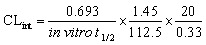


From these outcomes, FTB and LPB metabolic stabilities were characterized by a high CL_int_ (6.33 and 5.63 mL min^−1^ kg^−1^) and a low *in vitro t*_1/2_ (23.9 and 26.9 min). FTB metabolic rate is slightly decreased in combination with LPB, while LPB metabolic rate is greatly increased in combination with FTB (Table 6).

## 4. Conclusions

A validated LC-MS/MS methodology was developed for simultaneous estimation of FTB and LPB. The established method is highly sensitive, echo friendly (small volume of ACN), fast (run time = 5 min.), accurate, and has high recovery. The metabolic stability of FTB and LPB were characterized by a high CL_int_ (6.33 and 5.63 mL min^−1^ kg^−1^) and a low *in vitro t*_1/2_ (23.9 and 26.9 min), which revealed a high clearance of FTB and LPB from the blood by the liver. This probably resulted in a low *in vivo* bioavailability that corroborated the low oral bioavailability previously reported and also indicated that FTB and LPB will not bioaccumulate after multiple doses. FTB metabolic rate is slightly decreased in combination with LPB, while LPB metabolic rate is greatly increased in combination with FTB. So dose recalculation should be considered when FTB and LPB are used in combination.

## Conflicts of interest

There are no conflicts to declare.

## Authors' contributions

MAA, MWA, HAA, and HWD designed the study. MAA, HAA, and AAA supervised the practical work. AHB, MAA and MWA performed the optimization and method validation studies. MWA wrote the first version of the manuscript. All authors approved and revised the final version of the manuscript.

## Abbreviations

ACN: Acetonitrile
FTB: Foretinib
LPB: Lapatinib
CL_int_: Intrinsic clearance
CID: Collision-induced dissociation
ESI: Electrospray ionization
LC-MS/MS: Liquid chromatography tandem mass spectrometry
PI: Product ion
RLM: Rat liver microsomes
TKIs: Tyrosine kinase inhibitors
*t*_1/2_
: *In vitro* half-life
VEGFR: Vascular endothelial growth factor receptor-2
MET: Mesenchymal–epithelial transition factor
MST: Masitinib
PI: Product ion
LOD: Limit of detection
LOQ: Limit of quantification
MRM: Multiple reaction monitoring

## Supplementary Material
